# Supplementary material for: Impact of Polypharmacy for Chronic Ailments in Colon Cancer Patients: A Review Focused on Drug Repurposing
Source: Cancers (Basel). 2020 Sep 23;12(10):2724. doi: 10.3390/cancers12102724 (PMC7598185; doi:10.3390/cancers12102724)
Supplement: Supplementary file 1 [file cancers-12-02724-s001.pdf]

Supplementary Materials:

Impact of Polypharmacy for Chronic Ailments in Colon Cancer Patients: A Review Focused on Drug Repurposing

Riccardo Giampieri *, Luca Cantini, Enrica Giglio, Alessandro Bittoni, Andrea Lanese, Sonia Crocetti, Federica Pecci, Cecilia Copparoni, Tania Meletani, Edoardo Lenci, Alessio Lupi, Maria Giuditta Baleani and Rossana Berardi

Table S1. Summary of studies for drugs cited in the review, listed by effect on colorectal cancer incidence.

Drugs	Number of Patients	Results Favouring Drug	Results Not Favouring Drug	Results Against Drug	Citation No.
ALL antihypertensive	324,168	/	No difference in cancer incidence in users vs. not.	ACEi + ARB 10% RR increase.	Bangalore [9]
ARB/ACEi vs. others	138,769	/	No difference in CRC risk between ARB users vs. other	/	ARB trialists [10]
ARB users vs. others	111,533	/	No difference in CRC risk between ARB users vs. other. No difference for short- or long-term use.	/	Htoo [11]

CCBs users vs. others	23,167	/	No difference in CRC risk between CCBs vs. other.	/	Sorensen [12]
ALL antihypertensive	11,697 + 10× controls	/	No difference in CRC risk between users vs. not.	/	Assimes [13]
SSRI vs. others TCA vs. others	6544 for tryciclic<br>3367 for SSRI	SSRI users decreased risk of CRC	No difference in CRC risk between TCA users vs. other	/	Xu [69]
SSRI vs. others TCA vs. others	10,011 for tryciclic<br>2484 for SSRI	SSRI users decreased risk of CRC	No difference in CRC risk between TCA users vs. other	/	Coogan [70]
SSRI vs. others TCA vs. others	649 (+656 controls)	SSRI users decreased risk of CRC<br>TCA users decreased risk of CRC	/	/	Chubak [71]
TCA vs. others	31,953 + 2× controls	TCA users had decreased risk of CRC<br>The effect was cancer-specific	No difference in risk of other cancer types (lung, breast and prostate)	/	Walker [72]
ALL antidepressants	49,342 + 240,985 controls	Mirtazapine users decreased risk of CRC	/	MAOI users increased risk of CRC	Lee [74]

ALL antidepressants	9979 + 99,790 controls	/	No difference in CRC risk between users vs. not	/	Cronin-Fenton [75]
ALL antidepressants	418,588 + 1× controls	/	/	Small (not statistically significant) increased risk of CRC	Haukka [76]
ALL antidepressants	109,096 + 426,402 controls	/	No difference in CRC risk between users vs. not	Increased risk of lung and breast cancer for SSRI and TCA users	Boursi [77]
ALL antidepressants	2580 + 142,610 controls	Severe depressive symptoms associated with 20% risk of CRC	No difference in CRC risk between users vs. not	/	Kiridly-Calderbank [78]
Statins vs. nonusers	1953 cases + 2015 healty controls	Statin users decreased risk of CRC	/	/	Poynter [97]
Statins vs. nonusers	3244 cases + 14,844 controls	/	No difference in CRC risk between users vs. not	/	Kaye [98]
Statins vs. nonusers or users of other lipid-lowering drugs	12,251 statin users + 1257 users of other lipid-lowering drugs (population-based cohort study)	/	No difference in CRC risk between users vs. not	/	Friis [99]

HMG-CoA reductase inhibitors vs. users of bile acid–binding resins	542 cases + 5420 controls	/	No difference in CRC risk between HMG-CoA reductase inhibitors users vs. not	/	Blais [100]
Statins vs. nonusers	Meta-analysis of 18 studies	Modest reduction in CRC risk among case-control studies	No difference in CRC risk among RCTs and cohort studies	/	Bonovas [101]
Statins vs. nonusers	Meta-analysis of 42 studies	Modest reduction in CRC risk	/	/	Liu [102]
Statins vs. nonusers	Meta-analysis of 40 studies	Modest reduction in CRC risk among case-control and cohort studies	No difference in CRC risk among RCTs	/	Lytras [103]
Statins vs. nonusers	15,558 statin users + 75,597 nonusers (cohort study)	Reduction in the risk of rectal cancer	No difference in overall CRC risk between users vs. not	/	Lee [104]
Statins vs. nonusers (non-elderly population)	32,616 cases + 325,086 controls	Stronger effect of statins on CRC risk in participants aged 55 years or younger	/	/	Sehdev [105]
Statins vs. nonusers (patients with inflammatory bowel disease)	11,001 patients with inflammatory bowel disease (cohort study)	Reduction in CRC risk	/	/	Ananthakrishnan [106]

Statins vs. nonusers	25,811 cases + 129,117 controls	Reduction in the risk of rectal cancer	No difference in overall CRC risk between users vs. not	/	Ibáñez-Sanz [107]
ALL antibiotics	25 observational studies (n = 7,947,270 patients)	/	/	18% higher risk of cancer in antibiotics users (small increase in risk in CRC)	Petrelli [45]
ALL antibiotics	4029 cases and 15988 controls	/	/	Increase in CRC risk with penicillin and quinolone	Dik [53]
Oral antibiotics vs. not users	28,980 CRC cases and 137,077 controls identified	Decrease in rectal cancer risk with tetracyclines	/	Increase in colon cancer risk with penicillin and ampicillin/amoxicillin	Zhang [54]
Oral insulin secretagogues and insulin vs. not users	8194 incident cancer cases and 32,776 diabetic controls	/	/	Sulfonylureas and glinides increased the risk for overall cancer but to a lesser extent than insulin	Chang [81]
Metformin vs. not users	151 patients randomised	Reduced the prevalence and number of metacronous polyps	/	/	Higurashi [84]
ASA vs. not users	Review	Chemoprotective properties in CRC	/	Side effects	Lee [24]
NSAIDs vs. not users	Review	Protective effects on intestinal tumours	/	/	Couturier [25]
ASA vs. not users	Review	Protective effects on CRC PIK3CA-mutated patients	/	/	Grancher [26]
ASA vs. not users	Review	Beneficial effect against some types of cancer, particularly of the GI tract	/	/	Santilli [30]

Salicylic acid vs. not users		Reduction of incidence and mortality of CRC	/	/	Ai [31]
ASA vs. not users	Review	Reduction of long-term risk of some cancers, particularly CRC	/	/	Dovizio [32]
ASA vs. not users	Review	Reduction of risk of CRC and recurrence of adenomatous polyps	/	ASA-induced bleeding	Thun [33]
ASA and NA-NSAIDs	Meta-analysis of 23 studies (1,000,000)	Chemoprevention of CRC	/	/	Tomić [39]
